# Association between Physical Condition and Body Composition, Nutrient Intake, Sociodemographic Characteristics, and Lifestyle Habits in Older Spanish Adults

**DOI:** 10.3390/nu10111608

**Published:** 2018-11-01

**Authors:** Maria del Mar Bibiloni, Joanne Karam, Cristina Bouzas, Raquel Aparicio-Ugarriza, Raquel Pedrero-Chamizo, Antoni Sureda, Marcela González-Gross, Josep A. Tur

**Affiliations:** 1 Research Group on Community Nutrition and Oxidative Stress, University of the Balearic Islands, E-07122 Palma de Mallorca, Spain; mar.bibiloni@uib.es (M.d.M.B.); joannem.karam@gmail.com (J.K.); cristinabouvel@gmail.com (C.B.); tosugo@hotmail.com (A.S.); 2 CIBEROBN (Physiopathology of Obesity and Nutrition CB12/03/30038), 28029 Madrid, Spain; marcela.gonzalez.gross@upm.es; 3 Faculty of Health Sciences, University of Balamand, Balamand Al Kurah, P.O. Box 166378, Achrafieh, Beirut 1100-2807, Lebanon; 4 ImFINE Research Group, Department of Health and Human Performance, Faculty of Physical Activity and Sport Sciences (INEF), Technical University of Madrid, 28040 Madrid, Spain; apariciougarriza.raquel@gmail.com (R.A.-U.); Raquel.pedrero@gmail.com (R.P.-C.)

**Keywords:** physical condition, handgrip muscle strength, 8-foot time up-and-go, body composition, sarcopenia

## Abstract

In this study, we assessed physical condition and its association with body composition, nutrient intake, sociodemographic characteristics, and lifestyle habits in older Spanish adults. In this cross-sectional study, we investigated 380 individuals (54% women; men aged 55–80 years and women aged 60–80 years) with no previously documented cardiovascular disease. A general questionnaire was used, and body weight, height, fat, appendicular skeletal muscle mass, and waist circumference were assessed. Physical condition measurements included handgrip strength (HGS) and agility/dynamic balance by eight-foot time up-and-go (8-f TUG) test. The lowest maximum HGS score (kg) was found in older participants, inactive men, and men with abdominal obesity. The highest maximum 8-f TUG score (s) was found in older and inactive, low education, low income, and abdominal obesity and overfat participants; 24.5% of participants had low maximum HGS and 36.8% had a high 8-f TUG score. Sex- and/or age-adjusted odds ratio (OR) for low maximum HGS in women, older participants, overweight and overfat participants were 4.6, 2.9, 0.6 and 0.6 respectively. Sex and/or age adjusted OR for high maximum 8-f TUG in women, overweight, overfat, and abdominally obese participants were 2.4, 1.6, 1.7, and 3.4, respectively; in participants with higher education, those who earned €900 or more per month, and slightly active and active participants had OR values of 0.4, 0.4, and 0.3, respectively. Sarcopenia incidence was 0.3%; however, 4.5% of men and 19.1% of women registered low physical condition (high and low scores in 8-f TUG and HGS tests, respectively). Overall, 36.8%, 24.5%, and 0.3% of participants had high maximum 8-f TUG score, low maximum HGS, and sarcopenia, respectively. Prevalence of these low values varies according to sociodemographic and body composition variables.

## 1. Introduction

The current prevalence of and the expected increase in the elderly population is an important health challenge in our society. In 2050, almost 30% of the European population will be over 65 years old [1], and in Spain, it is estimated that 37% of the population will be over 64 years old by 2052 [2]. With an aging population, an increase in age-related diseases, including frailty, is expected.

Adverse health outcomes of aging, including falls, hospitalisation, institutionalization, and mortality, are the effect of impaired homeostatic reserve and reduced capacity of the organism to withstand stress, characterised by physical weakness, reduced physical activity, and performance that usually accompany frailty [3,4]. A key component of frailty is sarcopenia—a progressive loss of skeletal muscle mass and low muscle strength or performance that occurs with advancing age [4,5]. Sarcopenia is defined as a low muscle mass accompanied by either low muscle strength or low physical performance [4]. Low handgrip strength (HGS) and high eight-foot time up-and-go (8-f TUG) are used in the diagnosis of sarcopenia [4]; however, the prevalence of sarcopenia varies depending on the criteria used for diagnosis [6,7]. The European Working Group on Sarcopenia in Older People (EWGSOP) differentiated between presarcopenia (low muscle mass), sarcopenia (low muscle mass plus low muscle strength or physical condition), and severe sarcopenia (low muscle mass plus low muscle strength plus physical condition) [8].

Muscle strength has been described as a strong predictor of mortality and hospitalization in people aged 80 years and older [9]. Muscle homeostasis, under normal circumstances, is influenced by nutritional factors and level of physical activity [5], and also by chronic diseases and certain drug treatments [4]. Accordingly, moderate-to-vigorous physical activity may reduce the risk of severe sarcopenia and sarcopenic obesity among older men. Reducing sedentary time and increasing light physical activity and sedentary breaks may also protect against sarcopenic obesity [10]. Body composition and body measurements were shown to be associated with physical function; the Health, Aging, and Body Composition (Health ABC) study cohort reported poorer physical function in women due to their higher proportion of body fat compared to men [11]. In addition, increased body mass index (BMI) and waist circumference are associated with decreased cardiorespiratory fitness [12]; hence, a direct relation exists between BMI, waist circumference, body fat, and physical condition.

Early declines in physical condition can be detected through observation of a decline in the ability to perform mobility tasks activities of daily living, instrumental activities of daily living, or increased time to complete them. These symptoms need to reach a threshold before being recognized as a problem [13]. Early identification of a decline in the physical condition and appropriate interventions could help prevent functional impairments, such as impairments in walking and stairs climbing that often result in falls and physical frailty [14]. Therefore, the aim of this work was to assess physical condition, using HGS and 8-f TUG, and its association with nutrient intake, body composition, sociodemographic characteristics, and lifestyle habits in older Spanish adults.

## 2. Methods

### 2.1. Study Design, Population, and Ethics

The sample consisted of 380 participants (54% women) in a cross-sectional study conducted from 2013 to 2014 into two different areas (Balearic Islands, *n* = 211, and Madrid, *n* = 169) assessing the effect of lifestyle factors on health of older adults living in Spain [15]. Men aged between 55 and 80 and women aged between 60 and 80 [16] were recruited in social and municipal clubs, health centers, and sport clubs. Exclusion criteria included previously documented cardiovascular disease, being institutionalized, suffering from a physical or mental illness that would have limited participation in physical fitness or ability to respond for themselves to our questionnaires, chronic alcoholism or drug addiction, and intake of drugs for clinical research over the past year. The study was conducted according to the Declaration of Helsinki guidelines, and all procedures were approved by the Ethical Committee of the Technical University of Madrid. Written informed consent was obtained from all participants.

### 2.2. Body Composition

Anthropometric measurements (height, waist circumference (WC), appendicular skeletal muscle mass (ASM), body weight, and body fat) were recorded by licensed observers. Height and WC measurements were performed according to the International Standards for Anthropometric Assessment of the International Society for the Advancement of Kinanthropometry (ISAK) [17]. Height was determined using a mobile anthropometer (Seca 213, SECA Deutschland, Hamburg, Germany) to the nearest millimeter, with the subject’s head in the Frankfurt plane. WC was measured as the smallest horizontal girth between the costal margins and the iliac crests at minimal respiration using a flexible, non-extensible plastic tape with 0.1 cm precision (Kawe 43972, Kirchner & Wilhelm GmbH + Co., KG, Asperg, Germany). Body weight, body fat, and ASM were determined using a Segmental Body Composition Analyzer (Tanita BC-418, Tanita, Tokyo, Japan). One measurement was taken without excessive exercise 12 h before the measurement and/or no excessive eating and drinking the day before measurement. The participants were weighed in bare feet and light clothes, and subtracting 0.6 kg for their clothes. Weight and height measures were used to calculate body mass index (kg/m^2^). Further information may be found in the technical leaflets of this analyser [18]. According to the anthropometric reference parameters for the Spanish elderly [19,20], the prevalence of overweight and obesity was defined as BMI ≥ 27.0 kg/m^2^. Overfat (excessive body fat) was defined according to body fat ranges for Standard Adults reported by Gallagher et al. [21]. WC and height measures were used to calculate waist-to-height ratio (WHtR). Abdominal obesity was defined as a WHtR ≥ 0.5 [22]. ASM and height measures were used to calculate appendicular skeletal muscle mass index (ASMI, kg/m^2^) and low ASMI was defined as <7.26 kg/m^2^ in men and <5.5 kg/m^2^ in women [23].

### 2.3. Socioeconomic and Lifestyle Determinants

A specific questionnaire developed by the EXERNET network [24] that included the following questions was used: age, marital status, educational level, income, and smoking habits. The respondents were grouped in binary categories as follow: (a) age: <65 (men) and <67 (women) years old, and ≥65 (men) and ≥67 (women) years old; (b) marital status: single (single, unmarried, divorced, or widowed), and in a relationship (i.e., including married and unmarried, divorced or widowed living with a partner); (c) educational level: illiterate or primary (≤6 years), and secondary or college-level education (>6 years); (d) participants’ income: <€900 /month, and ≥€900/month; and (e) smoking habits: smoker (≥1 cigarette/day) and non-smoker.

Physical activity data were analyzed using the validated Spanish version of the Minnesota Leisure Time Physical Activity Questionnaire [25,26], and the participants were classified according to their leisure-time physical activity (LTPA) in the past 5 years. Individuals with ≤1.5 h/week of physical activity were categorised as “inactive”. Individuals who completed ≥4 h/week of physical activity were categorised as “active”. People who could not be included into the “inactive” and “active” groups were categorised as “slightly active”.

### 2.4. Physical Condition and Sarcopenia Definition

The physical condition assessment included muscle strength based on HGS and agility/dynamic balance based on the 8-f TUG test.

#### 2.4.1. Handgrip Strength Test (HGS) 

Grip muscular strength was measured using a digital handheld dynamometer (TKK 5401 Grip-D; Takey, Tokyo, Japan). Participants were instructed to stand upright with the dynamometer beside, but not against, their body. Measurements were performed two times for each hand. The best of all attempts was used to perform the analysis [27]. According to Cruz-Jentoft et al. [4], low handgrip strength was defined as <20 kg in women and <30 kg in men.

#### 2.4.2. Agility/Dynamic Balance Test (8-f TUG) 

Participants were instructed to rise from a chair without the use of arms, walk around the cone placed 2.45 m from the chair, and return to the original sitting position. Further instructions were to complete the test as quickly as possible but without running. Measurements were performed two times and the best of all attempts was used to perform the analysis [28]. Low execution time in the 8-f TUG test was defined using the cut-off points for age and sex presented in the study by Rikli and Jones. Since there is no cut-off for men aged 55–60 years we used the same cut-offs of men aged 60–64 years [29].

The algorithm provided by EWGSOP was adopted to determine whether the study individuals were sarcopenic [4]. Presarcopenia was defined as a low ASMI. Sarcopenia was defined as a low ASMI and either high 8-f TUG score or low HGS. Finally, severe sarcopenia was defined as a low ASMI combined with a high 8-f TUG score and low HGS [23].

### 2.5. Dietary Intake Assessment

Dietary intake was assessed by two non-consecutive 24 h recalls. Volumes and portion sizes were reported in natural units, household measures, or with the aid of a book of photographs [30]. Conversion of food into energy intakes and macronutrient content (carbohydrate, protein, total fat, polyunsaturated fatty acids (PUFA), monounsaturated fatty acids (MUFA), saturated fatty acids (SFA), cholesterol, and fiber) was completed using a self-made computerized program based on Spanish food composition table [31]. Energy intakes ranged between 1021 and 3515 kcal/day for men and 652 and 2996 kcal/day for women. Under-reporters (energy intake/basal metabolic rate <0.96) were 23.9% (50 men and 41 women) [32]; however, when macronutrient intake was compared between under-reporters and non-under-reporters, statistically significant differences were found only for protein in both sexes, and SFA and fiber in women. As such, under-reporters were not excluded from the present analysis.

### 2.6. Statistics

Analyses were performed with the SPSS statistical software package version 24.0 (SPSS Inc., Chicago, IL, USA). Maximum HGS and the maximum 8-f TUG test were calculated for men and women according to different sociodemographic, body composition, and lifestyle variables. The normality of the data was assessed using the Kolmogorov-Smirnov test. We found that dependent variables (maximum HGS and maximum 8-f TUG) were not normally distributed for sex and many sociodemographic, body composition, and lifestyle variables. Results are expressed as the median (interquartile range, IQR). The Mann-Whitney *U* test was used to compare the median of two independent groups, and the Kruskal-Wallis test was used to compare the median of three independent groups (i.e., LTPA physical variable). The differences in prevalence across normal and low maximum HGS, and normal and high maximum 8-f TUG score participants, according to sociodemographic, body composition, and lifestyle variables, were examined by using the Chi-square test. Logistic regression analyses, with the calculation of corresponding odds ratio (OR) and the 95% confidence interval (95% CI), were used to compare participants with low maximum HGS or high maximum 8-f TUG and the other participants as reference value (dependent variable) in selected sociodemographic, body composition, and lifestyle variables. Univariate analysis was first carried out for all the sociodemographic, body composition, and lifestyle variables (crude OR). Secondly, results were adjusted for sex and age to control for confounders. Results were considered statistically significant if *p* < 0.05 (two-tailed). Scatter plots were used to illustrate the correlations between the outcome measures used for sarcopenia diagnosis (HGS and 8-f TUG test). Analyses were stratified by sex.

## 3. Results

The maximum HGS and 8-f TUG score among Spanish older adults are shown in Table 1. Median maximum HGS was 37.2 kg (IQR: 11.1) in men and 21.8 kg (IQR: 6.0) in women. Median maximum HGS was lowest in men and women aged 65 and 67 or older, respectively. There were no significant differences in other sociodemographic variables. Men with abdominal obesity (*p* = 0.017) had lower maximum HGS. In men, significant differences in maximum HGS were also found by LTPA groups, with the lowest median maximum HGS in inactive men (35.6 kg, IQR: 6.2).

Median maximum 8-f TUG was 4.6 s (IQR: 1.1) for men and 5.2 s (IQR: 1.0) for women. Men older than 65 and women older than 67 had higher median maximum 8-f TUG score than younger participants. Whereas no significant differences in maximum 8-f TUG score were found in marital status, educational and income levels had an impact on 8-f TUG. Men and women with a primary education had higher maximum 8-f TUG score than high-education participants. Lower income was also associated with higher maximum 8-f TUG score for men and women. Participants with overweight, overfat, and abdominal obesity had higher median maximum 8-f TUG score than their leaner counterparts (results were significant except for BMI in men). Significant association with LTPA groups was found, with inactive men and women showing the highest median maximum 8-f TUG score.

Prevalence of normal and low maximum HGS and 8-f TUG score among Spanish older adults according to sociodemographic, body composition, and lifestyle variables is shown in Table 2. Overall, 24.5% of the participants had low maximum HGS. More women (36.8%) than men (10.2%) were below the cut-off values established for the HGS (*p* < 0.001). More men and women older than 65 and 67, respectively, showed low maximum HGS than younger counterparts (*p* < 0.001). Prevalence of low maximum HGS was higher in participants who were single (35.1%) and those with an income lower than €900 (33.1%) compared to married or coupled participants (20.8%) and those with an income of €900 or higher (19.8%) (*p* < 0.010). No significant differences in prevalence of low HGS and educational level were found. Overweight, overfat, and abdominally obese participants had lower prevalence of low maximum HGS than leaner counterparts. Despite the lack of statistical significance, participants who were slightly active and active showed lower prevalence of low maximum HGS than inactive participants (21.2% and 29.1%, respectively, *p* = 0.076).

Sex, age, marital status, income, overweight, overfat, and abdominal obesity status were significantly associated with low maximum HGS in univariate logistic regressions. Results in sex- and/or age-adjusted analysis illustrate that the odds of low maximum HGS were 4.6 (95% CI: 2.6–8.2) and 2.9 (95% CI: 1.7–4.8) times higher for women and older participants, respectively, compared with men and younger participants. In both overweight and overfat participants, the odds of low maximum HGS were 0.6 times lower for overweight (95% CI: 0.4–1.0) and overfat (95% CI: 0.3–1.0) participants compared with their leaner counterparts. However, the OR for low maximum HGS lost its statistical significant association with the income variable after adjusting for sex and age.

Conversely, 36.8% of participants had a high 8-f TUG score. More women (45.1%) than men (27.3%) were above the cut-off values established for the 8-f TUG test (*p* < 0.001). Prevalence of high 8-f TUG was higher in participants with primary education (48.4%) and an income lower than €900 (51.1%) than more educated participants (28.3%) and those with higher income (29.1%) (*p* < 0.001). More overfat (40.8%) and abdominal obese (41.0%) participants had a higher 8-f TUG score than leaner participants (30.3% and 22.4%, respectively, *p* < 0.050). Inactive participants also had a higher prevalence of high 8-f TUG than slightly active and active ones (*p* < 0.001). Age group, marital status, BMI status, and smoking habits were not significantly associated with 8-f TUG prevalence.

Sex, educational level, income, overfat, abdominal obesity, and LTPA were significantly associated with high maximum 8-f TUG in univariate logistic regressions. Results in sex- and/or age-adjusted analysis illustrated that the odds of a high maximum 8-t TUG were, respectively, 2.4 (95% CI: 1.5–3.7), 1.6 (95% CI: 1.0–2.5), 1.7 (95% CI: 1.1–2.7) and 3.4 (95% CI: 1.9–6.1) times higher for women, overweight, overfat, and abdominal obesity participants compared with the reference groups. In more educated participants, participants with an income of 900 euros or higher per month, and slightly active and active, the odds of a high maximum 8-f TUG were 0.4 (95% CI: 0.3–0.7), 0.4 (95% CI: 0.3–0.7), and 0.3 (95% CI: 0.2–0.5) times lower compared with the reference groups, respectively. Furthermore, educational and income levels maintained statistical significant association with high maximum 8-f TUG in multivariable analysis adjusted by sex and age (secondary or college: OR: 0.50, 95% CI: 0.32–0.80; income ≥ €900/month: 0.56, 95% CI: 0.34–0.94).

**Figure 1:**
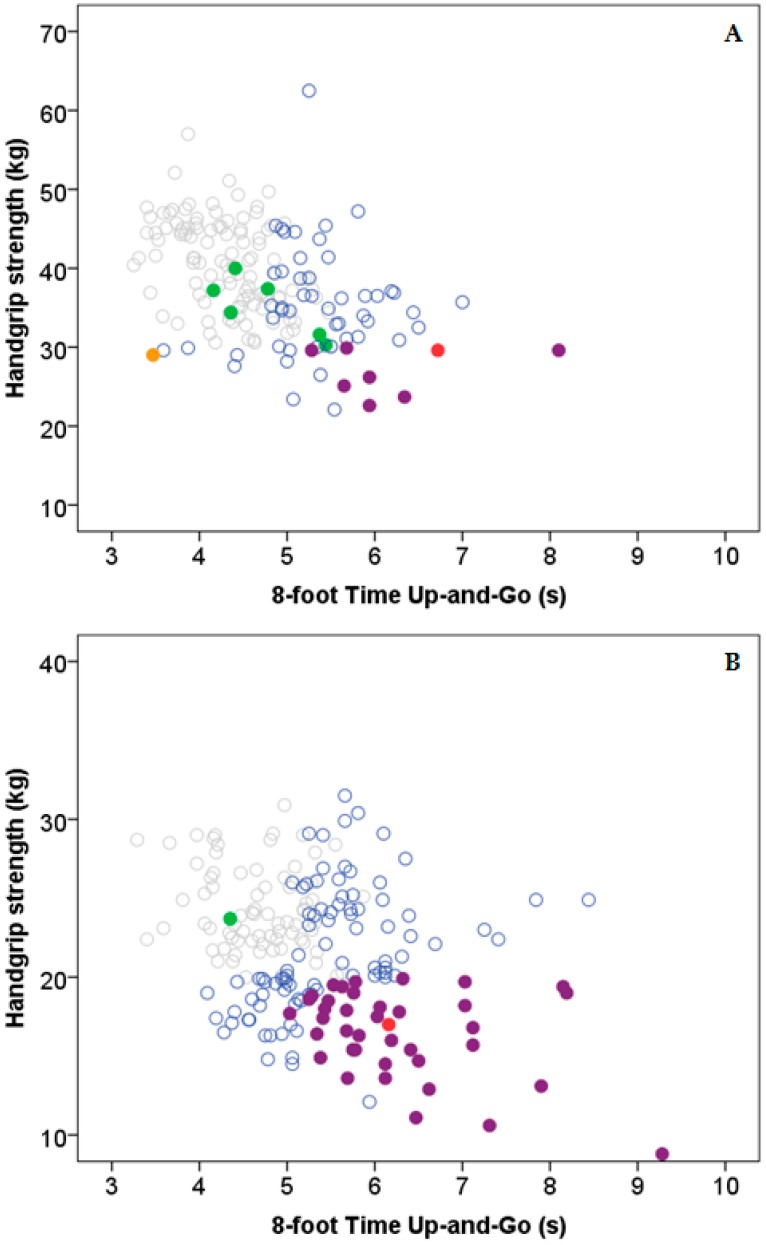
Scatter plot of variables used for diagnosis of sarcopenia: (**A**) men and (**B**) women. Grey open circles denote normal appendicular skeletal muscle index (ASMI, kg/m^2^), 8-foot Time Up-and-Go (8-f TUG, s), and handgrip strength (HGS, kg). Grey solid circles denote presarcopenia. Grey open triangles denote high 8-f TUG or low HGS. Grey solid triangles denote high 8-f TUG and low HGS. Black open squares denote sarcopenia, and black solid squares denote severe sarcopenia.

Figure 1 illustrates the correlation between 8-f TUG and HGS among men (Figure 1A) and women (Figure 1B). The frequency of presarcopenia was 2.6% (*n* = 10), and was more frequent in men (4.5%; *n* = 8) than women (1.0%; *n* = 2). The frequency of sarcopenia and severe sarcopenia was 0.3% (*n* = 1) and 0.5% (*n* = 2) in men and women, respectively. Eight men showed high 8-f TUG and low HGS scores (including severe sarcopenic participants), and 39 women registered high and low scores in both physical tests (*p* < 0.001).

Concerning macronutrients intake (Table 3), women with low HGS had significantly lower intake of calories, total fats, MUFAs, cholesterol, and fiber than women with low HGS, and significantly higher intake of carbohydrates. No differences between groups were observed for protein, PUFA, or SFA intake. No statistically significant differences between low/normal HGS groups were found in macronutrient intake in men. Additionally, no statistically significant differences between high/normal 8-fTUG groups were found in macronutrient intake in both sexes.

## 4. Discussion

The main findings of this study were that maximum HGS was lower among older participants, non-overweight women, inactive men, and men with abdominal obesity. Moreover, 24.5% of the participants had low maximum HGS and this risk increased in women, adults older than 65 and 67 for men and women, respectively, and decreased in overweight and overfat older adults. The maximum 8-f TUG score was higher among older participants, those with lower education, lower income, and inactive participants, those with abdominal obesity and overfat, and women with a BMI ≥27 kg/m^2^. Additionally, 36.8% of participants had high 8-f TUG score and this risk increased in women, and overweight, overfat, and abdominal obese participants; and decreased in highly educated participants, those with an income of €900 or more per month, and slightly active and active participants. Presarcopenia, sarcopenia, and severe sarcopenia prevalence was 2.6%, 0.3%, and 0.5% among the studied population, respectively. However, 4.5% of men and 19.1% of women registered low physical condition (assessed by high scores in 8-f TUG and low scores in HGS tests) but not low ASMI.

HGS for men was higher in this study than in a sample of Japanese men (*n* = 742) aged 70 ± 9 years (33.4 kg, SD: 7.5) from the Nomura study, but it was similar for women (*n* = 937) aged 70 ± 8 years (21.3 kg, SD: 4.1) [33]. In a cross-sectional analysis of the baseline data from a cohort study conducted in 2012 that included 1971 functionally-independent, community-dwelling Japanese adults aged 65 years or older (977 men, 994 women), HGS means were 34.8 kg (SD: 6.0) for men and 22.4 kg (SD: 3.9) for women [34]. The Hertfordshire Cohort reported different values of HGS among men (44.3 kg) and women (26.7 kg) older adults [35]. Patiño et al. [8] reported dissimilar HGS and 8-f TUG in community-dwelling persons over 60 years old from a northern Spanish city. These differences between studies are possibly due to difference in muscle strength among different populations [36] and to differences in healthy aging among different populations [37], reflected in the minor but existent differences in HGS.

The prevalence of low HGS (24.5%) and high 8-f TUG (36.8%) scores in this study was lower than reported in a northern Spanish city (13.2% and 13.6%, respectively). Compared to the Patiño et al. findings [8], in which participants with a low HGS had higher body fat (%) than participants with normal HGS, our study revealed a lower prevalence of low maximum HGS among those with a BMI ≥27 kg/m^2^ and those who showed abdominal obesity and overfat. However, similar to the Patiño et al. findings, in which participants with a high 8-f TUG score had higher body fat (%) than participants with normal test results, our study also revealed that overfat and abdominal obese participants had a higher prevalence of a higher maximum 8-f TUG score. This might be due to the fact that fat affects mobility and balance in older people [38]. A systematic review [39] concluded that although muscle and fat mass are considered important factors of age-related decline in physical function, studies examining the association between fat and muscle mass and functionality have produced inconsistent results.

The frequency of sarcopenia (0.3%) in this study was lower than in a northern Spanish city (2.4%), and unlike this northern Spanish city, women were more prone to being affected (19.1% of women registered low physical condition) [2]. This is reflected positively in the population studied, as the low frequency of sarcopenia implies a decreased risk of adverse health outcomes, including falls, loss of independence, and disability. As such, this decreases the socioeconomic burden in the studied population [40].

To the best of our knowledge, data on the association between sociodemographic characteristics and lifestyle habits, and physical condition in older adults are scarce. Physical activity has a major effect on physical condition [41]. Physical activity behavior is affected by two major components of socioeconomic status (SES): educational level and income. Educational level plays a primary role in the level of physical activity; an age-related decline in physical activity was observed among low-education individuals [42]. Lower education was associated with sarcopenia in the Korean National Health and Nutrition Examination Survey KNHANES [43] and in Invecchiare in Chianti (Aging in the Chianti area Study; InCHIANTI) [44]. Our results showed higher prevalence of high 8-f TUG among participants with lower education. Income plays a major role in determining he physical activity; individuals with higher income were more engaged in physical activity according to several studies [45,46]. Income was reported to be lower among those with sarcopenia [43]. In our study, prevalence of low HGS and high 8-f TUG scores was higher among those with a lower income, despite the lack of significance when OR was sex- and age-adjusted in HGS analysis. Our findings showed that inactive participants had higher 8-f TUG scores than active or slightly active participants. Notably, 8-f TUG assesses the agility and dynamic balance in older people, which is important in tasks that require quick accomplishment, such as alighting from a bus in time [14] that are easier to perform by active people. Finally, being single was also associated with higher prevalence of low HGS (despite the lack of significance when OR was sex- and age-adjusted), which agrees with studies that associated single relationship status with sarcopenia [47,48].

An adequate nutritional intake is an important element of any strategy to preserve muscle mass and strength during aging. Muscle wasting is a multifactorial process. A loss of fast twitch fibers, insulin resistance, glycation of proteins, and lipid deposition in muscle cells play important roles in the loss of muscle strength and development of sarcopenia [49,50]. Protein intake is crucial for muscle health and an intake of 1.0–1.2 g/kg of body weight per day is optimal for older adults [50]. High-fat diets may compromise aged muscle, diminishing overall muscle quality and composition [49]. Along this line, the findings of Charlton et al. [51] provide support regarding the importance of physical activity and adequate dietary protein intake for optimal body composition and the maintenance of strength and physical function. Sarti et al. [52] provided evidence that physical performance declines with advancing age, even in healthy women, and this decline in physical activity could lead to a lower intake of calories, carbohydrates, fats, and proteins. People, as they age, eat less and make different food choices. Lower food intake among the elderly has been associated with lower intakes of calcium, iron, zinc, B vitamins, and vitamin E [53]; this also negatively affects health.

### Strengths and Limitations

The main strength of this study is due to its strict protocol through validated measurement tools and the objective measurement of physical condition. The assessment method for sarcopenia according to the EWGSOP can be used in clinical practice and as a screening method in public health [54,55], which supports the reliability and accuracy of our results. Height and weight are frequently used to determine BMI and nutritional status in epidemiological research on older adults; however, BMI is an imperfect measure of overweight and obesity. Overfat and abdominal obesity (measured by WHtR that may be the single best clinical indicator of health risk) were also assessed in the present study [56]. 

However, this study has some limitations. Firstly, the present cross-sectional design limits the ability to elucidate a causal relationship between HGS and 8-f TUG scores and nutrient intake, body composition, sociodemographic characteristics, and lifestyle habits. Secondly, physical activity was not measured objectively, such as using an accelerometer, and sedentary leisure time activities as well as sleep habits were not measured either. Thirdly, height was measured using a stadiometer, which is the established gold standard, but this assessment may not be feasible for studies conducted in elderly populations with mobility limitations [57]. Fourthly, the macronutrient intake was estimated using recall diets instead of food frequency questionnaires that have been questioned in epidemiological studies [58,59]. Two 24-h recall diets tend to underestimate the food intake over a large period compared to food frequency questionnaires, and demonstrate a considerable day-to-day variation in macronutrient intake. Fifth, underreporting was calculated using energy intake/basal metabolic rate and medications types (e.g., antidepressants, influence weight, etc.) that might influence basal metabolic rate, and lifestyle factors and physical activity were not considered in the present study. Finally, the results potentially lack generalizability due to the selecting participants from only two sites as well as the sample size obtained.

## 5. Conclusions

Overall, 36.8%, 24.5%, and 0.3% of participants had high maximum 8-f TUG score, low maximum HGS, and sarcopenia, respectively. Additionally, 4.5% of men and 19.1% of women registered low physical condition assessed by high and low scores in 8-f TUG and HGS tests, respectively. Prevalence of low maximum HGS differed according to sex, age, weight, and fat status; and high maximum 8-f TUG differed according to sex, educational level, income, presence of extra weight, fat, and abdominal obesity, and LTPA practice. Strategies for early identification of decline in physical condition and appropriate interventions should be adopted to avoid physical impairments.

## Figures and Tables

**Table 1:** Maximum handgrip strength (HGS) and maximum eight-foot time up-and-go (8-f TUG) score among older adults according to sociodemographic, body composition and lifestyle variables stratified by sex.

Variable	*N*	Maximum HGS (kg)		Maximum 8-f TUG (s)	
	Men/Women	Men	Women	Men	Women
*Sociodemographics*					
All	176/204	37.2 (11.1)	21.8 (6.0)	4.6 (1.1)	5.2 (1.0)
Age (years)					
<65 (men)/67 (women)	83/96	40.9 (9.7)	22.7 (5.4)	4.4 (1.0)	5.0 (1.2)
≥65 (men)/67 (women)	93/108	36.3 (9.0)	20.3 (6.6)	4.9 (1.1)	5.3 (1.0)
*p*		<0.001	0.007	<0.001	0.003
Marital status					
Married/Coupled	153/130	37.2 (11.3)	22.0 (5.9)	4.7 (1.1)	5.3 (1.1)
Single	23/74	36.7 (7.8)	21.5 (6.5)	4.6 (1.3)	5.2 (1.0)
*p*		0.448	0.629	0.242	0.794
Educational level					
Primary	61/100	36.5 (9.7)	21.4 (6.3)	4.9 (1.1)	5.5 (1.1)
Secondary or college	115/104	38.1 (11.0)	22.2 (5.4)	4.6 (1.1)	5.0 (0.9)
*p*		0.124	0.398	0.011	<0.001
Income					
<900 €	27/106	36.3 (13.4)	21.9 (6.2)	4.9 (1.3)	5.4 (1.2)
≥900 €	149/98	37.4 (10.9)	21.8 (5.9)	4.6 (1.0)	5.0 (0.9)
*p*		0.253	0.662	0.040	<0.001
*Body composition*					
BMI (kg/m^2^)					
<27.0	74/113	36.8 (13.0)	21.4 (6.0)	4.4 (1.1)	5.1 (1.0)
≥27.0	102/91	37.5 (9.6)	22.5 (5.8)	4.8 (0.9)	5.4 (1.2)
*p*		0.989	0.202	0.128	0.007
Overfat					
No	59/83	41.1 (12.2)	20.4 (6.4)	4.3 (1.0)	5.1 (0.8)
Yes	117/121	36.7 (8.1)	22.5 (5.6)	4.8 (0.9)	5.3 (1.2)
*p*		0.063	0.143	<0.001	0.010
Abdominal obesity					
No	23/62	43.3 (10.2)	20.2 (6.3)	4.2 (0.9)	5.0 (0.7)
Yes	153/142	36.9 (10.5)	22.4 (5.9)	4.7 (1.0)	5.3 (1.0)
*p*		0.017	0.140	<0.001	0.002
*Lifestyle variables*					
Smoking habit					
Non-smoker	161/191	37.2 (11.3)	21.7 (5.5)	4.6 (1.1)	5.2 (1.0)
Smoker	15/13	37.4 (9.6)	22.9 (6.4)	4.7 (1.3)	5.2 (1.7)
*p*		0.836	0.216	0.717	0.652
LTPA					
Inactive	66/92	35.6 (6.2) ^a,b^	20.9 (6.2)	5.0 (1.0) ^a,b^	5.5 (1.1) ^a,b^
Slightly active	43/59	38.7 (10.7)	22.1 (6.3)	4.4 (1.0)	5.2 (0.9)
Active	67/53	40.9 (10.2)	22.4 (5.5)	4.4 (1.0)	4.8 (1.1)
*p*		0.001	0.310	<0.001	<0.001

Abbreviations: BMI, body mass index; LTPA, leisure-time physical activity. Values are expressed as median (interquartile range, IQR). Significant differences in maximum HGS and 8-f TUG medians between sociodemographic, body composition and lifestyle variables groups in men and women were tested by Mann-Whitney U test or Kruskal-Wallis test. Significant differences between (*p* < 0.05) ^a^ Inactive vs. Slightly active, and ^b^ Inactive vs. Active were obtained.

**Table 2:** Prevalence of low/normal maximum HGS and high/normal 8-f TUG score according to sociodemographic, body composition, and lifestyle variables.

Variables		Maximum HGS					Maximum 8-f TUG				
	*n*	Normal	Low	*p*	Crude OR (95% CI) ^†^	Sex- and/or Age-Adjusted OR (95% CI) ^‡^	Normal	High	*p*	Crude OR (95% CI) ^†^	Sex- and/or Age-Adjusted OR (95% CI) ^‡^
*Sociodemographics*											
All	380	287 (75.5)	93 (24.5)				240 (63.2)	140 (36.8)			
Sex											
Men	176	158 (89.8)	18 (10.2)	<0.001	1.00 (ref.)	1.00 (ref.)	128 (72.7)	48 (27.3)	<0.001	1.00 (ref.)	1.00 (ref.)
Women	204	129 (63.2)	75 (36.8)		5.10 (2.90–8.98)	4.59 (2.56–8.23)	112 (54.9)	92 (45.1)		2.19 (1.42–3.37)	2.38 (1.52–3.72)
Age (years)											
<65/67	179	151 (84.4)	28 (15.6)	<0.001	1.00 (ref.)	1.00 (ref.)	106 (59.2)	73 (40.8)	0.133	1.00 (ref.)	1.00 (ref.)
≥65/67	201	136 (67.7)	65 (32.3)		2.58 (1.56–4.25)	2.85 (1.68–4.82)	134 (66.7)	67 (33.3)		0.73 (0.48–1.10)	0.72 (0.47–1.10)
Marital status											
Married/Coupled	283	224 (79.2)	59 (20.8)	0.005	1.00 (ref.)	1.00 (ref.)	180 (63.6)	103 (36.4)	0.758	1.00 (ref.)	1.00 (ref.)
Single	97	63 (64.9)	34 (35.1)		2.05 (1.24–3.40)	1.08 (0.61–1.91)	60 (61.9)	37 (38.1)		1.08 (0.67–1.73)	0.90 (0.54-1.49)
Educational level											
Primary	161	119 (73.9)	42 (26.1)	0.531	1.00 (ref.)	1.00 (ref.)	83 (51.6)	78 (48.4)	<0.001	1.00 (ref.)	1.00 (ref.)
Secondary or college	219	168 (76.7)	51 (23.3)		0.86 (0.54–1.38)	1.22 (0.73–2.05)	157 (71.7)	62 (28.3)		0.42 (0.27–0.64)	0.43 (0.27–0.66)
Income											
<900 €	133	89 (66.9)	44 (33.1)	0.004	1.00 (ref.)	1.00 (ref.)	65 (48.9)	68 (51.1)	<0.001	1.00 (ref.)	1.00 (ref.)
≥900 €	247	198 (80.2)	49 (19.8)		0.50 (0.31–0.81)	1.00 (0.58–1.71)	175 (70.9)	72 (29.1)		0.39 (0.25–0.61)	0.44 (0.27–0.71)
*Body composition*											
BMI (kg/m^2^)											
<27.0	185	128 (69.2)	57 (30.8)	0.005	1.00 (ref.)	1.00 (ref.)	124 (67.0)	61 (33.0)	0.128	1.00 (ref.)	1.00 (ref.)
≥27.0	195	159 (81.5)	36 (18.5)		0.51 (0.32–0.82)	0.59 (0.35–1.00)	116 (59.5)	79 (40.5)		1.38 (0.91–2.11)	1.61 (1.04–2.49)
Overfat											
No	142	98 (69.0)	44 (31.0)	0.023	1.00 (ref.)	1.00 (ref.)	99 (69.7)	43 (30.3)	0.041	1.00 (ref.)	1.00 (ref.)
Yes	238	189 (79.4)	49 (20.6)		0.58 (0.36–0.93)	0.58 (0.34–0.98)	141 (59.2)	97 (40.8)		1.58 (1.02–2.46)	1.72 (1.10–2.72)
Abdominal obesity											
No	85	55 (64.7)	30 (35.3)	0.008	1.00 (ref.)	1.00 (ref.)	66 (77.6)	19 (22.4)	0.002	1.00 (ref.)	1.00 (ref.)
Yes	295	232 (78.6)	63 (21.4)		0.50 (0.30–0.84)	0.57 (0.31–1.03)	174 (59.0)	121 (41.0)		2.42 (1.38–4.23)	3.38 (1.87–6.09)
*Lifestyle variables*											
Smoking habit											
Non–smoker	352	263 (74.7)	89 (25.3)	0.193	1.00 (ref.)	1.00 (ref.)	222 (63.1)	130 (36.9)	0.898	1.00 (ref.)	1.00 (ref.)
Smoker	28	24 (85.7)	4 (14.3)		0.49 (0.17–1.46)	0.61 (0.19–1.95)	18 (64.3)	10 (35.7)		0.95 (0.43–2.12)	0.97 (0.43–2.19)
LTPA											
Inactive	158	112 (70.9)	46 (29.1)	0.076	1.00 (ref.)	1.00 (ref.)	77 (48.7)	81 (51.3)	<0.001	1.00 (ref)	1.00 (ref.)
Slightly active/Active	222	175 (78.8)	47 (21.2)		0.65 (0.41–1.05)	0.79 (0.47–1.32)	163 (73.4)	59 (26.6)		0.34 (0.22–0.53)	0.33 (0.21–0.52)

Abbreviations: BMI, body mass index; HGS, handgrip strength; 8-f TUG, 8-foot time up-and-go; LTPA, leisure-time physical activity; OR, odds ratio; CI, confidence interval. ^†^ Logistic regression analysis considering the effect of one explanatory variable. ^‡^ Logistic regression analysis considering the effect of one explanatory variable and adjusted for sex and age.

**Table 3:** Energy and macronutrient intake according to low/normal HGS and high/normal 8-f TUG score.

Variables	Maximum HGS			Maximum 8-f TUG		
	Normal (*n* = 287)	Low (*n* = 93)	*p*	Normal (*n* = 240)	High (*n* = 140)	*p*
*All (n = 380)*						
Energy intake (kcal/day)	1679 (1394–2046)	1547 (1269–1833)	0.007	1708 (1394–2043)	1559 (1277–1844)	0.004
Carbohydrate intake (% total energy)	44.7 (38.2–50.0)	47.3 (41.5–53.6)	0.003	44.9 (38.0–50.0)	46.6 (40.3–51.4)	0.201
Protein intake (% total energy)	16.0 (14.2–18.6)	16.1 (13.4–19.0)	0.738	16.2 (14.4–18.7)	15.8 (13.2–18.8)	0.149
Fat intake (% total energy)	35.0 (30.0–40.0)	32.5 (28.6–37.6)	0.025	34.3 (29.8–39.8)	34.0 (29.4–38.6)	0.520
PUFA (% total energy)	4.2 (3.5–5.1)	4.2 (3.5–5.7)	0.369	4.3 (3.5–5.4)	4.1 (3.3–5.0)	0.084
MUFA (% total energy)	16.0 (13.4–18.6)	14.0 (12.3–16.9)	0.001	15.8 (13.2–18.6)	15.1 (12.7–17.8)	0.254
SFA (% total energy)	10.0 (8.2–12.5)	9.6 (7.3–11.2)	0.058	9.8 (8.0–12.0)	10.1 (8.1–11.9)	0.600
Cholesterol intake (mg/1000 kcal)	261.9 (177.4–383.9)	211.1 (127.3–309.6)	0.054	253.8 (170.4–384.2)	245.7 (160.5–356.5)	0.993
Fiber intake (g/1000 kcal)	65.6 (51.2–82.5)	58.7 (45.3–71.2)	0.006	64.5 (51.3–82.3)	60.5 (45.1–78.1)	0.625
*Men (n = 176)*	*n* = 158	*n* = 18		*n* = 128	*n* = 48	
Energy intake (kcal/day)	1926 (1579–2255)	1789 (1527–1892)	0.068	1914 (1606–2202)	1817 (1507–2265)	0.528
Carbohydrate intake (% total energy)	43.8 (37.8–49.6)	43.6 (34.5–53.4)	0.914	44.2 (37.6–49.7)	43.6 (38.5–50.2)	0.847
Protein intake (% total energy)	15.7 (13.9–18.2)	16.0 (14.1–19.7)	0.461	15.9 (14.3–18.5)	14.9 (12.9–18.2)	0.092
Fat intake (% total energy)	34.8 (29.8–39.6)	32.5 (29.6–40.8)	0.907	34.1 (29.6–39.6)	35.6 (31.2–40.1)	0.361
PUFA (% total energy)	4.3 (3.5–5.1)	5.0 (3.6–6.3)	0.160	4.3 (3.5–5.3)	4.2 (3.6–5.0)	0.515
MUFA (% total energy)	16.1 (13.5–18.3)	14.9 (12.9–18.3)	0.646	16.0 (13.2–18.3)	16.0 (13.8–18.3)	0.740
SFA (% total energy)	10.2 (8.2–12.5)	9.5 (7.3–11.6)	0.470	9.8 (7.8–12.2)	10.6 (8.9–13.1)	0.059
Cholesterol intake (mg/1000 kcal)	149.6 (101.7–210.7)	193.4 (94.1–277.3)	0.372	141.2 (101.1–205.9)	158.0 (102.6–220.5)	0.570
Fiber intake (g/1000 kcal)	9.4 (7.2–12.2)	10.2 (7.9–13.8)	0.449	9.8 (7.6–12.7)	8.5 (6.8–10.1)	0.040
*Women (n = 204)*	*n* = 129	*n* = 75		*n* = 112	*n* = 92	
Energy intake (kcal/day)	1456 (1236–1721)	1515 (1235–1820)	0.486	1463 (1286–1774)	1486 (1193–1735)	0.525
Carbohydrate intake (% total energy)	46.3 (38.5–51.1)	48.3 (43.4–54.0)	0.018	46.3 (38.9–51.3)	47.4 (41.4–52.8)	0.265
Protein intake (% total energy)	16.7 (14.5–19.5)	16.1 (13.3–18.7)	0.155	16.7 (14.6–19.1)	16.1 (13.4–19.1)	0.374
Fat intake (% total energy)	35.2 (30.3–40.3)	32.5 (28.5–36.8)	0.016	34.7 (30.2–40.2)	33.6 (29.0–37.7)	0.147
PUFA (% total energy)	4.1 (3.4–5.2)	4.2 (3.4–5.3)	0.700	4.3 (3.5–5.5)	4.0 (3.3–5.1)	0.106
MUFA (% total energy)	15.8 (13.4–18.7)	13.7 (12.2–16.5)	0.001	15.6 (13.2–18.6)	14.7 (12.4–17.2)	0.132
SFA (% total energy)	10.0 (8.3–12.1)	9.6 (7.3–11.2)	0.158	9.9 (8.2–11.5)	9.8 (7.7–11.2)	0.567
Cholesterol intake (mg/1000 kcal)	159.6 (119.6–232.0)	123.2 (91.4–179.6)	0.004	147.0 (111.0–202.2)	142.3 (92.9–227.1)	0.613
Fiber intake (g/1000 kcal)	10.9 (8.5–13.6)	11.1 (9.6–14.4)	0.159	11.1 (9.0–13.3)	10.6 (9.0–14.5)	0.779

Abbreviations: HGS, handgrip strength; 8-f TUG, 8-foot time up-and-go. Values are expressed as median (interquartile range, IQR). Significant differences in nutrient medians between low and normal HGS and 8-f TUG score were tested by Mann-Whitney *U* test.

## Availability of Data and Materials

The data analyzed in this paper can be made available to researchers. Requests for access to the dataset used in this paper should be directed to the corresponding author.

## Abbreviations

ASMI	appendicular skeletal muscle index
BMI	Body mass index
CI	Confidence interval
EWGSOP	European Working Group on Sarcopenia in Older People
8-f TUG	8-foot time up-and-go test; HGS: handgrip strength test
ISAK	International Society for the Advancement of Kinanthropometry
LTPA	Leisure-time physical activity
OR	Odds ratio
SPSS	Statistical Package for the Social Sciences
WHO	World Health Organization
